# Cryptococcal Tongue Lesion in a Stem Cell Transplant Patient: First Reported Case

**DOI:** 10.1155/2012/517415

**Published:** 2012-07-17

**Authors:** David R. Reinstadler, Sanjeet Dadwal, Ellie Maghami

**Affiliations:** ^1^Department of Otolaryngology-Head and Neck Surgery, University of Southern California, 1200 N State Street, Los Angeles, CA 90033, USA; ^2^Division of Infectious Disease, City of Hope National Medical Center, 1500 East Duarte Road, Duarte, CA 91010, USA; ^3^Division of Otolaryngology-Head and Neck Surgery, City of Hope National Medical Center, 1500 East Duarte Road, Duarte, CA 91010, USA

## Abstract

A compromised immune system places individuals at a significantly higher risk for many infectious processes. Immunosuppression also increases the risk of malignancy due to the body's decreased ability to perform its normal immunosurveilance and response. It is therefore imperative to have regular thorough evaluations of these patients, as slight abnormalities may be the early signs of infection or neoplasm. We present the first reported case of a tongue lesion in a stem cell transplant patient, highly concerning for malignancy, which was found to be a mucocutaneous presentation of disseminated Cryptococcus.

## 1. Introduction

Opportunistic infections pose a constant threat to the immunocompromised individual. Immunosuppression is encountered in the setting of hematopoietic stem cell transplantation (HSCT), solid organ transplantation (SOT), adult immunodeficiency syndrome (AIDS), neoplastic disease, metabolic disease, immunosuppressive therapy, malnutrition, or even advanced age. In the transplant population, fungi have emerged as important pathogens due to underlying net state of immunosuppression, extensive use of broad-spectrum antibiotic, and prolonged use of intravascular catheters. 

*Cryptococcus neoformans* is an opportunistic fungus that may affect different organ systems and therefore has a wide spectrum of clinical presentations. Inhalation of the aerosolized pathogen is the primary mode of transmission, and infection commonly spreads to the central nervous system (CNS) [1]. Subsequent cutaneous involvement results from hematologic dissemination [2] or less commonly direct cutaneous inoculation [3]. There is sparse literature characterizing this infection in the HSCT patient population, though the SOT literature quotes skin or soft tissue involvement in 17.8% of cases upon presentation, usually involving the lower extremities [4]. Oral Cryptococcal lesions are exceedingly rare with only a few reported cases in the setting of human immunodeficiency virus infection [5–7], and a single additional case of nonimmunocompromised patient with oral involvement [8]. To our knowledge, oral cryptococcal lesion has not yet been described in an HSCT patient. 

## 2. Report of a Case

Our patient is a 65-year-old gentleman who first presented to the neurosurgical department at the City of Hope for evaluation and treatment of a lytic lesion involving his thoracic spine causing compressive symptoms. Upon further workup, lytic lesions were noted throughout his cervical and thoracic spine, as well as a rib lesion. The patient underwent surgical decompression and debulking of the largest thoracic mass which was histologically consistent with a plasmacytoma. Bone marrow biopsy confirmed plasma cell myeloma. He underwent induction chemotherapy followed by radiation to his spine and subsequent autologous hematopoietic stem cell transplantation which was completed October 2010. His posttransplant course was complicated by fever, mucositis, gastroenteritis, and pneumonia, from which he recovered. 

He was diagnosed with persistent/relapsed multiple myeloma, and put on treatment with lenalidomide and dexamethasone in February 2011. In early May 2011, he was treated for pneumonia with multiple antibiotics and antifungal agent with subsequent improvement. He had tested negative for cryptococcus by serology and bronchoscopic alveolar lavage culture and cytologic examination. He was also negative for other pathogens.

In the later part of May 2011, he presented to clinic with an erythematous nodular rash, which started on his upper extremities and then spread to his face and scalp associated with peripheral eosinophilia, concerning for drug rash. Antibiotics were stopped, and he was started on steroids. He was seen multiple times over the next few weeks with varying degrees of this same rash; each time steroids were adjusted providing some improvement. In July of 2011 the patient was hospitalized for the persistent rash and a new painful tongue ulcer. Again, high-dose steroids were given along with antibiotics, and he was discharged within a week, improved. In September of 2011 the patient presented to the walk-in clinic with severe occipital headache, hypertension with a diastolic pressure above 100 mm Hg, and nausea/vomiting. A computed tomography (CT) scan of his head showed no discrete mass or lesion, but diffuse cerebral volume loss. Initially, the headaches improved with antihypertensive medication, but he was subsequently admitted for management of acute renal failure, persistent headache, and management of the tongue ulceration. 

**Figure 1:**
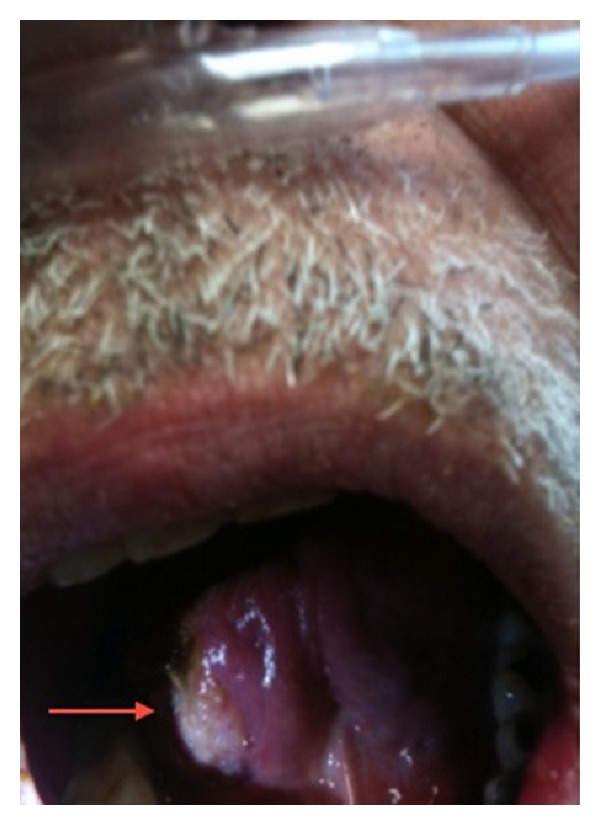
A 1.5 × 1.5 cm ulceration with slightly heaped boarders on the right lateral anterior oral tongue.

An otolaryngology consult was requested for evaluation of the oral ulceration. The patient was a poor historian. Upon questioning, he reported that this specific lesion had been present for only a week, but because it was not significantly painful, he could not be certain that it had not been present longer. He reports having smaller previous tongue lesions that resolved spontaneously. The ulceration only irritated him when eating hot, spicy, or acidic food. He was a social drinker and a former 20-pack year smoker, though he quit just prior to his diagnosis of myeloma. Physical exam revealed 1.5 × 1.5 cm ulceration with slightly heaped boarders on the right lateral anterior oral tongue (Figure 1). This ulceration was slightly tender but not friable or bleeding. The lesion was relatively superficial with no significant deep invasion and little surrounding erythema. The remainder of his head and neck physical exam was normal. There was no palpable cervical lymphadenopathy. There were multiple 1-2 mm umbilicated lesions on his chest, and a diffuse, scaly, and erythematous rash on his lower extremities and torso. He had no focal neurologic findings. 

**Figure 2:**
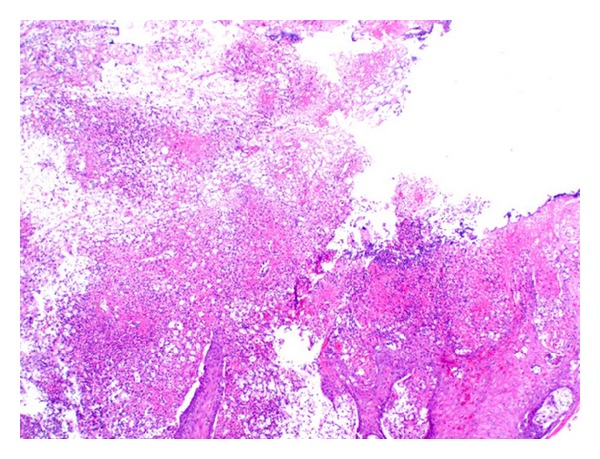
Low-power (40x) view of the lesion with surface ulceration, acute, and chronic inflammation.

**Figure 3:**
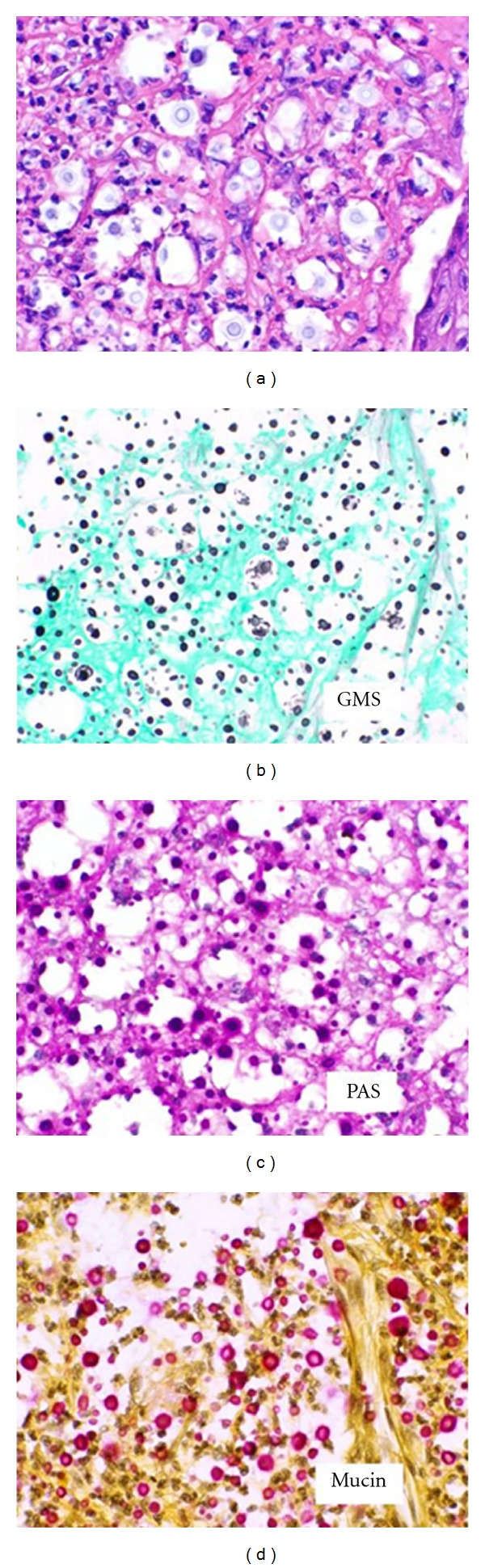
High-power (400x) view of the lesion with special stains for GMS, PAS, and mucin.

A biopsy of the tongue lesion was performed from the leading edge, including a portion of the heaped boarder and surrounding mucosa. The majority was sent to the pathology department with a small piece sent to microbiology. The final pathologic diagnosis showed squamous mucosa with marked acute and chronic inflammation along with numerous fungal organisms described as budding yeast, which stained positive for Gomori-Grocott methenamine silver (GMS), Periodic acid-Schiff (PAS), and mucicarmine (Figures 2 and 3). These findings were suggestive of *Cryptococcus neoformans* infection, and this was confirmed by tissue culture.

Upon identification of fungal organisms by histology, antifungal treatment was promptly initiated. Serum cryptococcal antigen tested positive and a lumbar puncture was performed which was also positive for cryptococcal antigen at a titer >1 : 32. Cryptococcus was cultured from the cerebrospinal fluid. Blood cultures grew the same. The high-dose antifungal therapy continued, but he became more confused over the next few days and was subsequently transferred to the intensive care unit and intubated for respiratory failure. A repeat CT scan was performed, which showed mild hydrocephalus and a lumbar drain was placed. Within the next 24 hours, the patient decompensated and became obtunded with fixed pupils. An emergent CT scan of his brain showed diffuse cerebral edema with impending herniation. Patient then became hypotensive requiring pressor support. After a family meeting, the patient was made “do not resuscitate” (DNR). He was terminally extubated and shortly thereafter he expired.

## 3. Comments

The incidence of invasive fungal infections has increased over the recent years [9] with the incidence of fungal sepsis increasing by 207% between 1979 and 2000 [10]. The risk of an invasive fungal infection in the HSCT recipient is higher in allogeneic transplant patients compared to autologous transplant patients [11]. Those with graft-versus-host disease, heavy use of corticosteroids, or leukopenia are also at an increased risk [11]. 

The type of invasive fungal infection varies based on the underlying condition. In a review by Pfaller and Diekema [9], patients with hematologic malignancies have the highest incidence of *Candida* species infections (42.6%) followed by *Aspergillus* (33.8%), Zygomycetes (5.2%), and *Cryptococcus* (2.1%). In a prospective study (TRANSNET) of hematopoietic stem cell transplant recipients, invasive aspergillosis (43%) was the leading cause of invasive fungal infection followed by invasive candidiasis (28%), and zygomycosis (8%) [12]. Though Cryptococcus accounts for only a small proportion of invasive fungal infections, the mortality rate remains high at 21% in this population [13]. This high mortality rate is likely due to early dissemination and CNS involvement. In the SOT patients who presented with cutaneous disease, 88.9% developed meningoencephalitis indicating probable disseminated disease at the time of presentation [4]. When the history of our patient was retrospectively analyzed, his symptoms prior to admission of a diffuse rash, headache, nausea, and vomiting may have been attributable to systemic fungemia, though they all resolved with relatively conservative measures; thus they did not raise significant suspicion. 

Secondary malignancies in stem cell transplant patients are of significant concern. A large review of this population estimated a cumulative risk for solid tumor development of 1.6% at 5 years, 6.1% at 10 years, and 14.9% at 15 years posttransplant [14]. The relative risk of oral squamous cell carcinoma (SCC) in this population is between 9.5–11.1 times that of the general population [14, 15]. With this significant increase in oral SCC, a thorough oral cavity and oropharyngeal evaluation needs to be performed when any suspicious lesions arise. A low threshold for biopsy should be taken into this examination.
